# Long-term survival after sequential local treatments for oligometastatic esophageal squamous cell carcinoma: A case report

**DOI:** 10.1016/j.ijscr.2022.107423

**Published:** 2022-07-19

**Authors:** Tiuri E. Kroese, Peter S.N. van Rossum, Sylvia van der Horst, Stella Mook, Nadia Haj Mohammad, Jelle P. Ruurda, Richard van Hillegersberg

**Affiliations:** a Department of Surgery, University Medical Center Utrecht, Utrecht University, Utrecht, the Netherlands; b Department of Radiation Oncology, University Medical Center Utrecht, Utrecht University, Utrecht, the Netherlands; c Department of Medical Oncology, University Medical Center Utrecht, Utrecht University, Utrecht, the Netherlands

**Keywords:** Esophageal cancer, Oligometastasis, Metastasectomy, OMD, oligometastatic disease, OS, overall survival, 18F-FDG PET/CT, ^18^F-fluorodeoxyglucose positron emission tomography with integrated computed tomography, EUS, endoscopic ultrasound, RAMIE, robot-assisted minimally-invasive esophagectomy, OMEC, OligoMetastatic Esophagogastric Cancer

## Abstract

• Oligometastatic disease (OMD) is associated with favorable tumor biology.
• Local treatment for OMD may improve overall survival (OS).
• Treatment for OMD is highly complex and requires a multidisciplinary team.

## Abstract (de)

**Introduction and importance:**

Patients with metastatic esophageal cancer generally have a poor prognosis. In patients with oligometastatic disease (OMD), local treatment of metastases may improve overall survival (OS).

**Case presentation:**

This case report describes a 57-year-old female with a cT4bN2M1 mid-esophageal squamous cell carcinoma with ingrowth in the aorta and pathologic para-aortic lymph node metastases in the left thorax (i.e. synchronous OMD). The patient first underwent a robot-assisted thoracoscopic para-aortic lymph node dissection (R1, aorta). After response to definitive chemoradiotherapy (ycT4aN1), a salvage robot-assisted minimally-invasive esophagectomy was performed (ypT0N1, R0). During follow-up, metastasectomy and repeated cryoablations for pulmonary metastases were performed (repeat OMD). The patient is currently alive in very good condition without evidence of disease 4.5 years after the first diagnosis of OMD and free of systemic therapy.

**Discussion:**

Clinical decision-making, local treatment options, and favorable biological tumor behavior in OMD are discussed.

**Conclusion:**

In this case of oligometastatic esophageal squamous cancer with favorable biological behavior, sequential local treatment of OMD (i.e. metastasectomy and cryoablations) combined with primary tumor resection was associated with long-term OS.

## 1 Introduction

Approximately 35 % of patients with esophageal cancer present with (synchronous) metastatic disease [1]. Metastatic esophageal cancer is associated with a poor prognosis, with a median overall survival (OS) of 8 months despite standard systemic therapy [2]. However, in patients with a limited number of metastases and favorable tumor biology, so-called oligometastatic disease (OMD), improved outcomes have been reported [3], [16]. The concept of OMD implies that local treatment for OMD may improve the time to disease progression and ultimately OS [4]. The European Society for Radiotherapy and Oncology (ESTRO) and European Organisation for Research and Treatment of Cancer (EORTC) published recommendations on the characterization and classification of OMD in 2020 [5]. A history of polymetastatic disease before diagnosis of OMD was used as a criterion to differentiate between induced OMD (i.e. history of polymetastatic disease) and genuine OMD (i.e. no history of polymetastatic disease). Subsequently, genuine OMD was sub-classified into repeat OMD (i.e. history of OMD) and de-novo OMD (i.e. first-time diagnosis of OMD). Finally, de-novo OMD was classified into synchronous and metachronous OMD [5]. This case report is described in accordance with the SCARE guidelines (Supplementary File 1) [6].

## 2 Case report

**Fig. 1:**
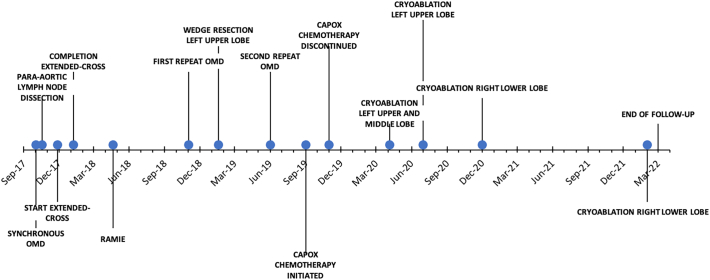
Timeline with milestones in diagnosis and treatment.

**Fig. 2:**
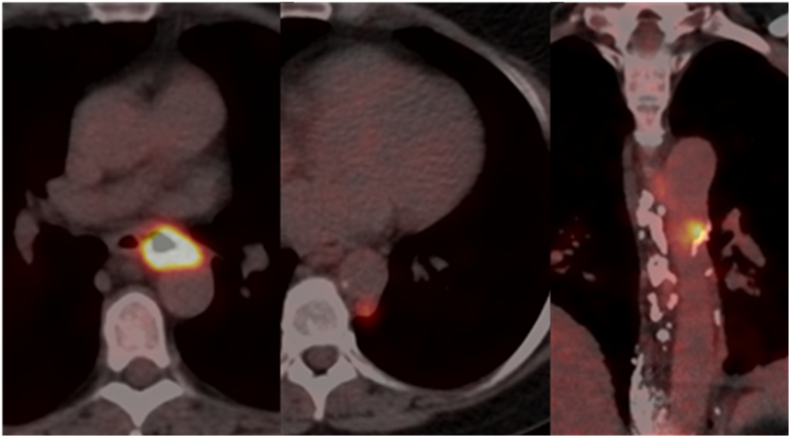
A ^18^F-FDG PET/CT scan of a 57-year-old female with a cT4bN2M1 mid-esophageal squamous cell carcinoma with ingrowth in the aorta and left para-aortic lymph node metastases.

This case report describes a 57-year-old female in good clinical condition with mid-esophageal squamous cell carcinoma (at 27–32 cm distance from incisors). The patient was clinically staged with esophagogastroscopy and ^18^F-fluorodeoxyglucose positron emission tomography with integrated computed tomography (^18^F-FDG PET/CT) as cT4bN2M1 (synchronous OMD; Fig. 1 [5]). Ingrowth in the aorta of the primary tumor and 2 para-aortic lymph node lymph metastases was confirmed with endoscopic ultrasound (EUS). The 2 suspected para-aortic lymph node metastases were located in the left thorax and showed pathologic FDG-uptake. A timeline with milestones of diagnosis and treatment is presented in Fig. 2.

### 2.1 Synchronous OMD

**Fig. 3:**
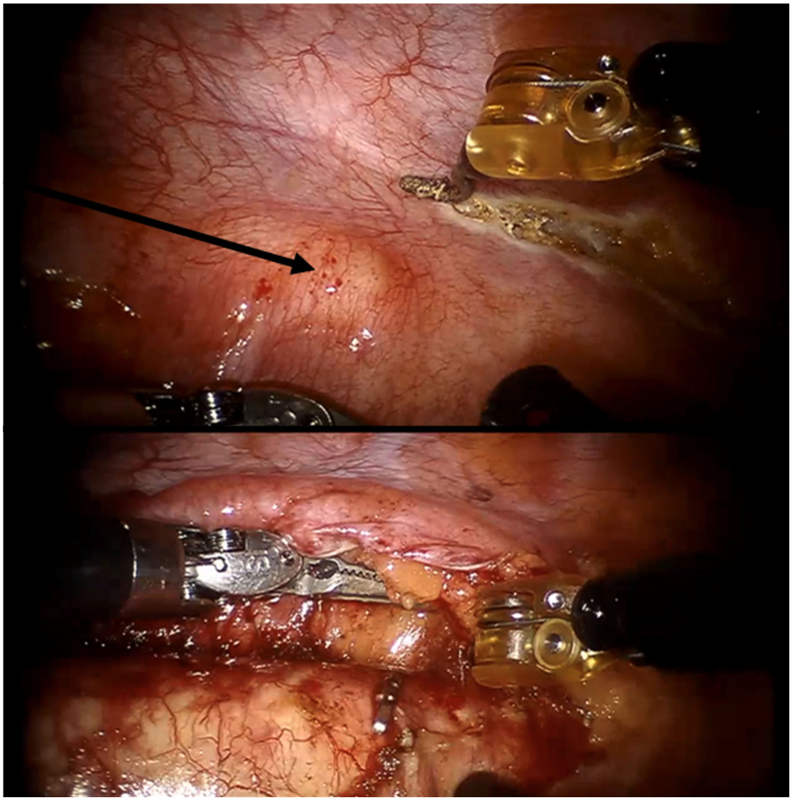
Intraoperative image of an en-bloc robot-assisted para-aortic lymph node dissection in the left thorax. The patient lies in right lateral semi-prone position and the camera is in the 6th right intercostal space. The pleura is incised at the lateral side of the descending aorta (just caudal of the arcus aortae) and at the dorsal side at the spine. The arrow indicates the suspected lymph node metastases.

The patient first underwent a robot-assisted thoracoscopic para-aortic lymph node dissection in the left thorax (Fig. 3). Pathologic examination demonstrated squamous cell carcinoma nodal metastasis with extra-nodal growth in 1 out of 3 resected lymph nodes. The resection was classified as R1 because of microscopic tumor involvement of the aortic resection margin. The patient started with extended-CROSS chemoradiotherapy (50.4 Gy radiotherapy in 28 fractions with concurrent weekly carboplatin/paclitaxel chemotherapy [7]). The radiotherapy clinical target volume (CTV) consisted of the primary tumor, the regional lymph nodes, and the R1 aortic resection margin. Treatment-related toxicity included dysphagia grade 3, for which total parenteral nutrition was initiated (complicated by central line infection). Six weeks after completion of extended-CROSS chemoradiotherapy restaging ^18^F-FDG PET/CT and EUS were performed. At restaging, the patient was clinically downstaged to ycT4aN1M0 because the primary tumor and regional lymph node metastases showed response to chemoradiotherapy (i.e. no tumor ingrowth in the aorta anymore and only 1 regional lymph node remained suspected for metastasis). In addition, no new (distant) metastases were detected.

Subsequently, 14 weeks after completion of extended-CROSS chemoradiotherapy a salvage robot-assisted minimally-invasive esophagectomy (RAMIE) and two-field regional lymphadenectomy with gastric tube reconstruction was performed (complicated by grade 3 anastomotic leakage requiring surgical drainage [8]). The resected specimen revealed a pathological complete response and radical resection of the primary tumor as well as 1 regional lymph node metastasis out of 38 resected regional lymph nodes (ypT0N1, Mandard 1, R0). No adjuvant treatment was given.

### 2.2 First repeat OMD

**Fig. 4:**
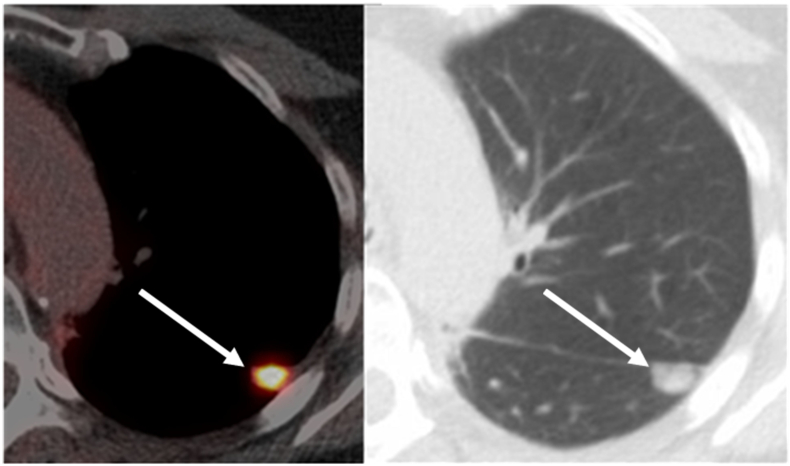
A ^18^F-FDG PET/CT scan 6 months after RAMIE demonstrating a 2.0 cm pulmonary metastasis in the left upper lobe with pathologic FDG uptake (indicated by the arrow).

At 6 months follow-up after RAMIE (1 year after primary tumor diagnosis), a 2.0 cm solitary FDG-avid pulmonary metastasis was detected in the left upper lobe (repeat OMD, Fig. 4). The pulmonary metastasis was successfully removed through video-assisted thoracoscopic wedge resection of the left upper lobe (complicated by grade 3 empyema requiring surgical drainage). Pathologic examination showed squamous cell carcinoma metastasis with a maximum diameter of 2.0 cm, which was radically resected (R0).

### 2.3 Second repeat OMD

**Fig. 5:**
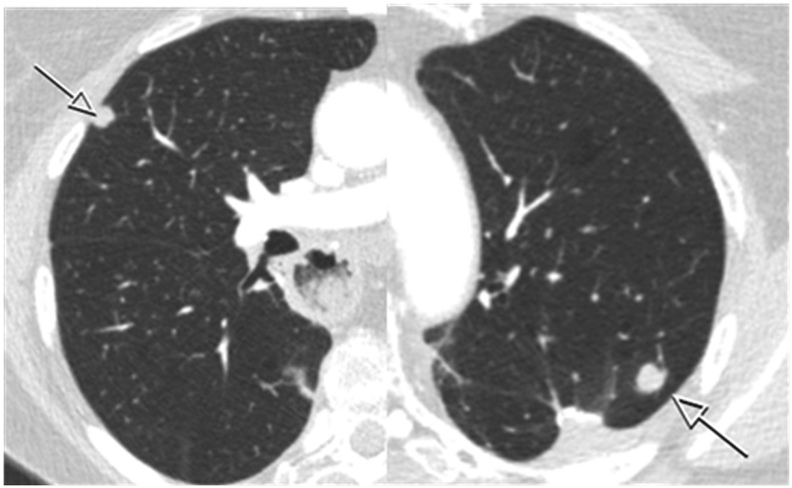
A CT scan 12 months after RAMIE demonstrating a 7.0 mm pulmonary metastasis in the right middle lobe and a 7.0 mm pulmonary metastasis in left upper lobe (indicated by the arrows).

At 14 months follow-up after RAMIE (18 months after primary tumor diagnosis), a total of 3 new FDG-avid pulmonary metastases were detected in the left upper lobe (7 mm), middle lobe (7 mm), and right lower lobe (3 mm, repeat OMD; Fig. 5). The physician and patient refrained from systemic therapy because of the low tumor load. After a 3-months therapy-free interval, the pulmonary metastases had slowly progressed in size (from 7 to 9 mm, from 7 to 8 mm, and from 3 to 5 mm, respectively). Capecitabine and oxaliplatin (CapOx) chemotherapy was initiated. A restaging CT after 3 cycles of CapOx chemotherapy demonstrated partial response of the pulmonary metastases according to RECIST criteria [9]. Chemotherapy was discontinued after 5 cycles because of grade 2 fatigue and grade 2 dyspnea.

At 2 years follow-up after RAMIE (2.5 years after primary tumor diagnosis), cryoablation of the 2 pulmonary metastases in the left upper lobe and middle lobe was performed (without complications). Cryoablation of the pulmonary metastasis in the right lower lobe was not performed because the lesion was considered too small for successful treatment (5 mm). Stereotactic body radiotherapy (SBRT) of pulmonary metastases was discussed, but considered too risky for the lungs because the recent radiotherapy already had a mean lung dose of 17 Gy. Subsequently (after a 2 months therapy-free interval after the previous cryoablations), 1 new pulmonary metastasis was detected in the right upper lobe and cryoablation of this metastasis was performed (without complications). After a therapy-free interval of 4 months, the (remaining) pulmonary metastasis in the right lower lobe had slowly progressed in size (from 5 to 9 mm), and the patient underwent cryoablation of this metastasis 2.5 years after RAMIE (without complications). Cryoablation of this pulmonary metastasis in the right lower lobe was repeated 4 months later (3 years after RAMIE) because the lesion had progressed (without complications).

### 2.4 Survival

Currently, the patient is alive in good clinical condition 4 years after RAMIE and 4.5 years after the primary tumor diagnosis (according to EQ-5D: some problems with walking, no problems with self-care, unable to perform usual activities, and moderate pain) and good psychosocial condition. Recent follow-up PET/CT imaging of the chest and abdomen detected no evidence of disease.

## 3 Discussion

This case report demonstrates that in a patient with oligometastatic esophageal squamous cell carcinoma with favorable tumor biology, sequential local treatment of OMD combined with primary tumor resection and short-term systemic therapy were associated with long-term OS. The favorable tumor biology is demonstrated by 3 factors.

First, it is demonstrated by the response of the primary tumor. At initial staging, the patient was diagnosed with tumor ingrowth in the aorta at the level of the primary tumor (i.e. cT4b) and at level of the para-aortic lymph node metastases. After extended-CROSS chemoradiotherapy, the primary showed good response to chemoradiotherapy, leading to tumor downstaging without ingrowth in the aorta (i.e. ycT4a). This made surgical resection possible with RAMIE. Subsequently, the pathologic specimen showed a pathologic complete response of the primary tumor and radical resection (ypT0, R0). Second, the response of the regional lymph node metastases is another sign of favorable tumor biology since pathologic lymph node metastases are the most important prognostic factor for OS in esophageal cancer patients who undergo chemoradiotherapy and esophagectomy [10]. At initial staging, the patient was diagnosed with 5 regional lymph node metastases (cN2). After extended-CROSS chemoradiotherapy and RAMIE, the pathologic specimen showed only 1 pathologic regional lymph node metastasis (outside the radiation field, ypN1). Third, the pulmonary metastases responded to CapOx chemotherapy and subsequently in 2 consecutive therapy-free intervals (of 3 months and 4 months, respectively) untreated pulmonary metastases showed only small progression in size. Indeed, response to systemic therapy is an important prognostic factor for OS in the first-line palliative setting for patients with metastatic esophagogastric cancer [11], although postponing systematic treatment could also have been a valid option which was discussed with the patient.

In addition, this case report demonstrates the value of an experienced multidisciplinary team, since it is difficult to gain experience in this field because oligometastatic esophagogastric cancer is relatively uncommon and complex. In order to improve the quality of care, treatment of OMD could be centralized in high-volume centers. In addition, a multidisciplinary European effort to improve the quality of care of patients with esophagogastric OMD is known as the OligoMetastatic Esophagogastric Cancer (OMEC) project (www.OMECproject.eu). OMEC is a consortium of 50 esophagogastric cancer expert centers in 15 countries in Europe and aims to achieve a multidisciplinary European consensus statement of the definition, diagnosis, and treatment of oligometastatic esophagogastric cancer. The OMEC project consists of 5 substudies, including a systematic review on definitions of OMD in esophagogastric cancer (OMEC-1) [12], real-life clinical case discussions by 50 cancer expert centers asking for multidisciplinary team responses on definition and treatment (OMEC-2) [13], and Delphi consensus process study through 2 online Delphi questionaire rounds and a consensus meeting (OMEC-3). Subsequently, the intended consensus statement for oligometastatic esophagogastric cancer (OMEC-4) is expected to pave the way for a clinical prospective study (OMEC-5).

Another approach to improve the quality of care of patients with OMD is studied in the ongoing phase III Renaissance (FLOT-5) trial by Arbeitsgemeinschaft Internistische Onkologie (AIO) [14]. This trial randomizes patients with gastric cancer with synchronous OMD limited to 1 organ and/or the retroperitoneal lymph nodes who respond to chemotherapy to either resection of the primary tumor and metastases or continuation of chemotherapy alone [14]. Furthermore, the ongoing phase II trial by Nguyen et al. randomizes patients with esophageal or gastric cancer with metachronous OMD limited to 3 metastases who respond to chemotherapy to either SBRT or continuation of chemotherapy alone [15].

Strengths of this case report include the extensive follow-up and good documentation of treatments for OMD as well as the intraoperative images of surgery. A potential limitation is that, inherent to a case description, it is unknown how the disease would have progressed without the resection of the para-aortic lymph node metastases and metastasectomy and repeated cryoablations of the pulmonary metastases.

## 4 Conclusion

In selected cases with metastatic esophageal cancer with favorable tumor biology such as described in the current report, sequential local treatment of OMD combined with primary tumor resection and systemic therapy are associated with long-term OS. Treatment of OMD is complex, requires an experienced multidisciplinary team, and might benefit from centralization in high-volume centers to improve quality of care.

## Provenance and peer review

Not commissioned, externally peer-reviewed.

## Source of funding

Not applicable.

## Ethical approval

The need for ethical approval was waived by the institutional review board.

## Consent

Written informed consent was obtained from the patient for publication of this case report and accompanying images. A copy of the written consent is available for review by the Editor-in-Chief of this journal on request.

## Author contribution

Study concept: TK, PvR, and RvH.

Data collection: TK, PvR, and RvH.

Data analysis or interpretation: all authors.

## Research registration

Not applicable.

## Guarantor

The authors.

## Declaration of competing interest

Not applicable.

## Appendix A Supplementary data

Supplementary material Image 1
